# Cœliotomy, Not Laparotomy

**Published:** 1892-11

**Authors:** 


### "CŒLIOTOMY," NOT "LAPAROTOMY."

---

Dr. Robert P. Harris, of Philadelphia, called attention two years ago to the inappropriateness of the term "laparotomy." *Lapara*, from which the word is derived, was applied in the original Greek to the parts between the short ribs and the crest of the ilium (the flank), and therefore the operation of lumbar colotomy would be a true laparotomy. This incorrect use of the word began with a Wittenberg student who coined it in 1811.

In a recent widely circulated pamphlet Dr. Harris again suggests the abandonment of the term and the adoption of "cœliotomy" as the correct substitute. In the original Greek it is *Koilia*, that means *belly*, and so it appears ten times in the Greek Testament; never *lapara*. *Koilia* was so employed by Rufus, of Ephesus, physician and writer of the second century. For correctness sake "cœliotomy" would therefore seem preferable to the word now in universal use. We already have *Cœlio-paracentesis* for tapping the abdomen. The new word has been adopted by Prof. Sanger, of Leipzig, by Dr. Greig Smith, in his *Abdominal Surgery*, and by Profs. Keene and White in their *American Text-Book of Surgery*.

Except for strict etymological accuracy we do not see that the new term has any special advantage over the old. Anatomical nomenclature is rich in such mis-applications which may never be corrected.

